# A pharmacokinetic–pharmacodynamic model based on multi-organ-on-a-chip for drug–drug interaction studies

**DOI:** 10.1063/5.0011545

**Published:** 2020-07-23

**Authors:** Kenta Shinha, Wataru Nihei, Tatsuto Ono, Ryota Nakazato, Hiroshi Kimura

**Affiliations:** 1 Department of Mechanical Engineering, School of Engineering, Tokai University, 4-1-1 Kitakaname, Hiratsuka, Kanagawa 259-1292, Japan; 2 Micro/Nano Technology Center (MNTC), Tokai University, 4-1-1 Kitakaname, Hiratsuka, Kanagawa 259-1292, Japan

## Abstract

In drug discovery, the emergence of unexpected toxicity is often a problem resulting from a poor understanding of the pharmacokinetics of drug–drug interactions (DDI). Organ-on-a-chip (OoC) has been proposed as an *in vitro* model to evaluate drug efficacy and toxicity in pharmacology, but it has not been applied to DDI studies yet. In this study, we aim to evaluate whether organ-on-a-chip technologies can be applied to DDI studies. To assess the usefulness of OoC for DDI studies, we proposed a multi-organ-on-a-chip (MOoC) with a liver part as the metabolic model and a cancer part as the drug target model, and a pharmacokinetic–pharmacodynamic (PK–PD) model describing the MOoC. An anticancer prodrug, CPT-11, was used to evaluate the drug efficacy of the metabolite in the liver part of the MOoC. To evaluate DDI using the MOoC, the inhibitory effect of simvastatin and ritonavir on the metabolism of CPT-11 was tested. The DDI estimation method was evaluated by comparing the results of the concomitant administration experiment using the MOoC and the results of simulation using the proposed PK–PD model with the estimated parameters. The results were similar, suggesting that the combination of the PK–PD model and the MOoC is a useful way to predict DDI. We conclude that OoC technologies could facilitate a better understanding of pharmacokinetic mechanisms with DDI.

## I. INTRODUCTION

New medicines are approved and manufactured following basic research, nonclinical tests, and clinical tests. The efficacy and toxicity of drug candidate compounds are evaluated in both nonclinical and clinical tests. However, toxicities not observed in clinical trials may appear after the medicines are sold. For example, serious side effects of the combination of sorivudine and 5-fluorouracil were reported in the 1990s. These unexpected toxicities are due to drug–drug interactions (DDI) caused by the concomitant administration of multiple drugs.^1^ Countless combinations of new drugs with other drugs are impossible to be evaluated during pre-production testing. Therefore, a detailed understanding of pharmacokinetics with DDI is necessary to reduce the risks associated with drug combinations.^2^ However, due to species differences between experimental animals and humans, experimental animals cannot adequately reproduce the reactions in humans. Therefore, it is difficult to accurately predict and understand the pharmacokinetics of drug–drug interactions in the human body through animal testing.^3^

To address this limitation, pharmacokinetic (PK) models have been utilized. The prediction of pharmacokinetics *in vivo* is made possible by mathematical models of the variability of drug concentration over time through absorption, distribution, metabolism, excretion, and accumulation in tissues and association with metabolic enzymes. Furthermore, efficacy and toxicity can be predicted effectively from the drug dose by combining PK and pharmacodynamic (PD) models (PK–PD), thereby representing the relationship between drug concentration and its effect.^4^ A PK–PD model is established based on drug-specific parameters obtained from *in vitro* tests using cultured cells and microsomes and then evaluated by comparing with the results of animal and clinical tests.^5^ However, the detailed evaluation of pharmacokinetics remains challenging because data from animal tests are discrete, and it is difficult to monitor the efficacy and drug concentration continuously. Furthermore, data from clinical tests are limited to concentrations of the compounds and metabolites in blood or excreta. Regarding *in vitro* tests using cultured cells, *in vivo* environments are not sufficiently reproduced, and the co-culture of cells of multiple organ models is challenging. Therefore, novel cell-based assay systems that complement conventional *in vitro* tests and animal tests are essential for the accurate prediction and increased understanding of pharmacokinetics of DDI.^6^

Microfluidics-based *in vitro* culture models such as organ-on-a-chip (OoC) and microphysiological systems have recently been considered as novel cell-based assay systems for pharmacokinetic research.^7–9^ To evaluate organ interactions *in vitro*, several organ model cells are cultured in different compartments connected by microchannels on OoC. Parameters of OoC, such as flow ratio between organ models, residence time of circulating medium in the organ parts, and ratio of the cell number to medium volume, accommodate the PK model by the design of microchannels.^4^ Therefore, OoC may be useful to evaluate the PK model and examine drug-specific physiological effects.^10^ The usefulness of combining the PK model and OoC has been demonstrated by the consistency between the drug concentration calculations obtained from the PK model and the experimental results obtained using OoC.^11^ In addition, the calculated parameters using OoC were more similar to those of *in vivo* tests than to those of conventional *in vitro* tests.^12^ The combination of the PK model and OoC has also been useful for research involving not only drugs but also metabolites, such as glucose, in the body.^12^ However, previous studies have not undertaken the study of concomitant administration with multi-drugs using OoC; thus, the usefulness of DDI studies using PK models and OoC has yet to be shown.

In this study, we aim to assess whether OoC technologies can be applied to DDI studies. To evaluate the usefulness of OoC for DDI studies, we proposed a multi-organ-on-a-chip (MOoC) with a liver part as the metabolic model and a lung cancer part as the drug target model, and a PK–PD model describing the MOoC. An anticancer prodrug, CPT-11, was used to evaluate the drug efficacy of the metabolite in the liver part of the MOoC. Drug-specific parameters were estimated by using the proposed PK–PD model from the results of drug efficacy tests by varying the flow ratio to the liver part and lung cancer part in the MOoC. To evaluate DDI using the MOoC, the inhibitory effect of simvastatin (SV) and ritonavir (RTV) on the metabolism of CPT-11 was tested. The DDI estimation method was evaluated by comparing the results of the concomitant administration experiment using the MOoC and the results of simulation using the proposed PK–PD model with the estimated parameters. In the present study, we demonstrated that the combination of the PK–PD model and the MOoC is a useful way to predict DDI.

## II. MATERIALS AND METHODS

### A. Development of the MOoC

**FIG. 1.**
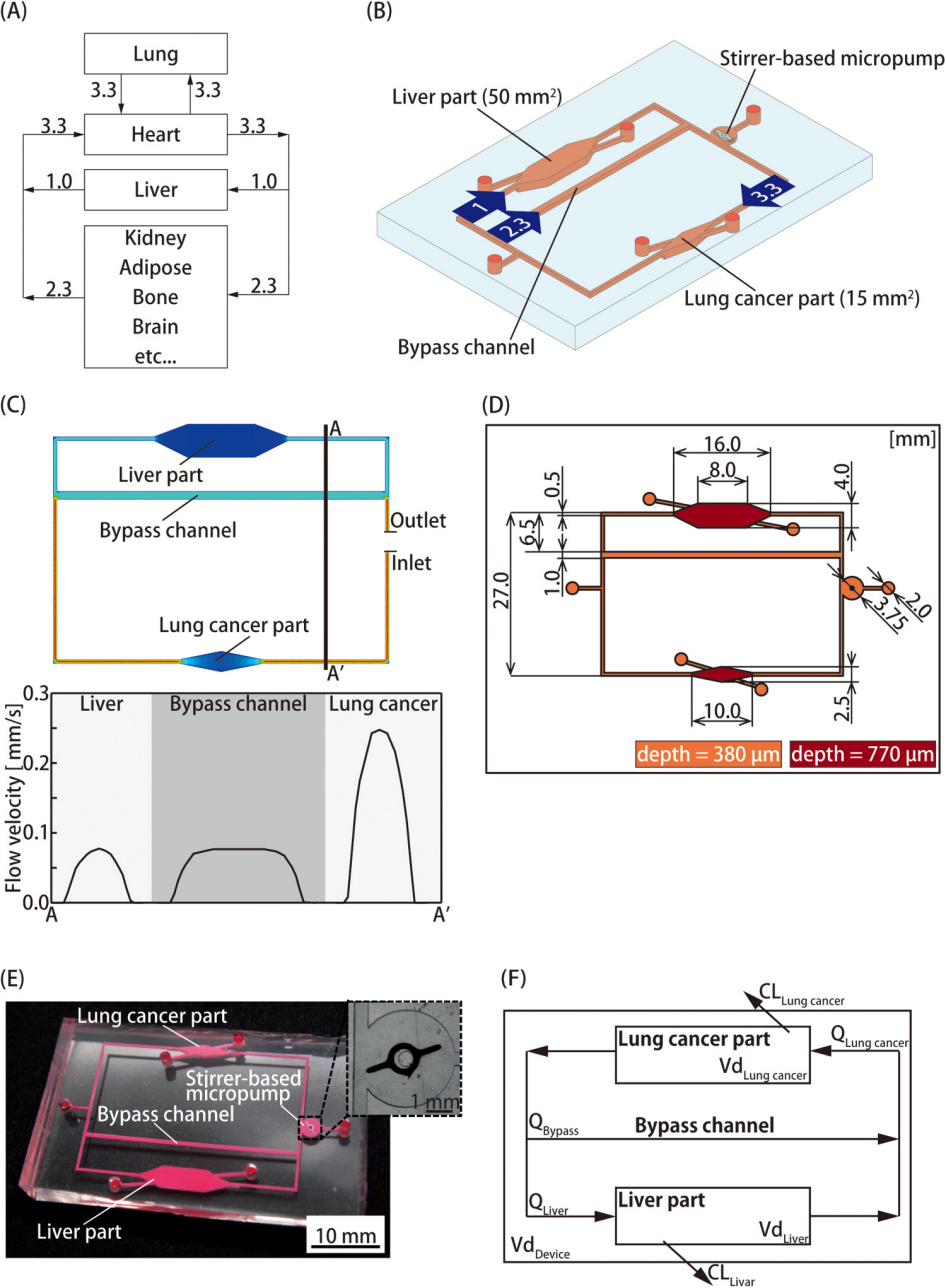
Pharmacokinetic–pharmacodynamic (PK–PD) model based on multi-organ-on-a-chip (MOoC) for evaluating drug–drug interactions. (a) Schematic of the blood flow ratio *in vivo*. After flowing into the lung, the blood is distributed to the organs by the heart. The flow ratio of the liver to the lung and heart is 1:3.3. (b) Schematic illustration of the MOoC, which consists of a liver part, a lung cancer part, and a stirrer-based micropump connected by microchannels. The values inside the arrows are the flow rate ratios. (c) Flow velocity in the MOoC as estimated by ANSYS 17.1. The graph shows flow velocity on A–A' cross section. (d) The dimensions of the microchannel on the MOoC. (e) Photograph of the fabricated MOoC with the enlarged view of the stirrer-based micropump as an inset. (f) Conceptual diagram of the PK model based on the MOoC. The change in drug concentration is dependent on the flow rate and extraction ratios of organ models.

We developed a MOoC integrated with a liver part having metabolic functions based on pharmacokinetics and a lung cancer part as a drug target. The blood *in vivo* is dispensed to the organs by the heart after assimilating oxygen from the lungs. The blood flow ratio between the liver, heart, and lung is 1:3.3:3.3 [Fig. 1(a)].^13^ A stirrer-based micropump, which we had developed previously, was integrated for medium perfusion of the MOoC.^14^ The flow ratio was controlled by the resistance of a bypass channel installed onto the MOoC to resemble *in vivo* conditions [Fig. 1(b)].

The design of the bypass channel was optimized using ANSYS 17.1 (ANSYS Inc., USA), a fluidic simulation software, with the finite element method [Fig. 1(c)]. The dimensions of the microchannel are shown in Fig. 1(d). The dimensions of the chambers of the lung and the liver cancer parts were 15 mm^2^ and 50 mm^2^, respectively. The heights of the channel and cell culture chambers were 380 *μ*m and 770 *μ*m, respectively. The cell culture chambers had step structures to avoid the leakage of cells.

The MOoC was fabricated by conventional photolithography and soft lithography methods.^15^ A master mold of PDMS chips was fabricated using a negative photoresist (SU-8 2100, MicroChem Corp., USA). A chromium mask for photolithography was designed using a computer-aided design software (AutoCAD, Autodesk, USA) and fabricated using a micro-pattern generator (*μ*PG101, Heidelberg Instruments Inc., Germany). The first layer of SU-8 for microchannels was spin-coated at 750 rpm for 15 s onto a silicon wafer. After baking, the microchannel was patterned on the substrate using an ultraviolet lamp. The second layer for the step structures of the cell culture chambers was fabricated similarly. Then, the SU-8 layers on the silicon wafer were developed and rinsed with MicroChem's SU-8 Developer and isopropanol, respectively. The substrate was used as a master mold for polydimethylsiloxane (PDMS; SILPOT 184, Dow Corning Toray, Japan), the material of the MOoC. A 10:1 mixture of PDMS and a polymerization agent was poured onto the master mold and heat cured in an oven at 75 °C for 2 h. The cured PDMS was peeled from the master mold to produce a PDMS chip to which the channel pattern was transferred. The surface of the PDMS was coated with CYTOP (CTL-107MK, AGC, Japan) to spin the stirrer bar smoothly. A 7% CYTOP solution was cast onto the micropump chamber and baked at 65 °C for 30 min and 100 °C for 60 min. The PDMS surfaces were activated using a plasma cleaner (PDC-32G, Harrick Plasma, USA). After the stirrer bar was set into the chamber, the MOoC was assembled to permanently bond to the PDMS chips [Fig. 1(e)].

### B. Flow characterization of the MOoC

Flow velocity was measured by the particle tracking velocimetry (PTV) method using fluorescent microbeads with a diameter of 1.17 *μ*m (17687, Polysciences, USA). The fluorescent microbeads were suspended in ultra-pure water with 0.1% Tween20 (103168, MP Biomedicals, USA). After the microchannels were filled, the suspension was perfused by the stirrer-based micropump at 1300–2800 rpm. The flowing microbeads in the microchannels were observed by a high-speed camera (MEMRECAM HX-3, nac Image Technology Inc., Japan) installed in a fluorescence microscope (IX71, Olympus, Japan). The flow rates in each organ were calculated from the velocities of the flowing fluorescent beads measured using ImageJ.

### C. Cell culture

HepG2 (JCRB1054, JCRB Cell Bank, Japan) was used as a liver model cell for metabolic functions, whereas A549 (RCB0098, RIKEN BRC, Japan) was used as a cancer model cell. The cells were cultured at 37 °C in an incubator in a humidified atmosphere containing 5% CO_2_. Dulbecco's modified Eagle medium supplemented with 10% fetal bovine serum (FBS, Bio West, Japan), 1% non-essential amino acid solution (11140–050, Thermo Fisher, USA), and 1% antibiotic antimycotic solution (161–23181, FUJIFILM Wako, Japan) was used as the culture medium for both cells.

Cell seeding and cell culturing were performed within the MOoC according to the following procedure. The cell culture chambers in the MOoC were coated with collagen type I-P (634-00663, Nitta Gelatin Inc., Japan) as an extracellular matrix (ECM). The collagen was chemically bonded to PDMS through 3-aminopropyltriethoxysilane (aminosilane, KBE-903, Shin-Etsu Chemical Co., Ltd., Japan) and glutaraldehyde (GAD; 17026-32, Kanto Chemical Co., Inc., Japan) coated on the bottom surface of the cell culture chambers.^16^ After the permanent bonding of PDMS chips, the cell culture chambers were coated with aminosilane by immediately removing the aminosilane after its introduction and allowing to stand at 54 °C for 2 h. Then, 2.5% GAD was introduced to the microchannel and allowed to stand at room temperature for 1 h. Finally, the microchannel was filled with 0.1 mg/ml collagen type I-P and allowed to stand at 4 °C for 12 h. The microchannel was washed with ultra-pure water after coating with aminosilane and GAD and with phosphate-buffered saline (PBS) after collagen coating. HepG2 cells were seeded into the liver part chamber at 1.7–2.0 × 10^5^ cells/cm^2^. After prior culture for 48 h, the A549 cells were seeded into the lung cancer part chamber at 1.7–2.0 × 10^4^ cells/cm^2^ and cultured for 24 h. The culture medium in the MOoC was exchanged daily.

### D. Establishment of the PK–PD model

A PK–PD model was established by combining a separately constructed PK model and a PD model. The PK model is a mathematical model showing time-dependent changes in the concentrations of drug and biological matter, whereas the PD model shows the relationship between drug efficacy and drug concentration. The PK–PD model is used to predict the drug efficacy on the target site using the drug dosage. However, alternative indices of drug concentration are needed in models involving drug concentration changes over time because the PD model expresses drug efficacy as a function of the drug concentration. Normally, the area under the concentration–time curve (AUC) is used as an alternative index to drug concentration and is an integral value of a drug concentration–time function as it represents the full drug dose *in vivo* in pharmacokinetics studies.

We established a PK–PD model that predicts the drug efficacy on the lung cancer part from values of the AUC obtained by the PK model. The microchannel volume of the MOoC and changes in the drug concentration in the organ parts must be considered because pharmacokinetics depends on the drug concentration in each organ part and the metabolism of compounds by these organs [Fig. 1(e)]. Only the metabolism of the liver part was considered in the proposed model because the number of metabolic enzymes in the lung cancer part, i.e., the drug target part, was considerably lower than that of the liver part. The gradient of the prodrug concentration is expressed as follows:

$$\frac{d[C_{P}]}{dt}=-k_{P}[C_{P}],$$ (1)

where *k_P_* is the elimination rate constant of the prodrug. The change in prodrug concentration is as follows:

$$C_{P}[t]=X_{0}\times e^{-k_{P}\times t},$$ (2)

where *X_0_* is the initial concentration of the prodrug.

The concentrations of the metabolites simultaneously increase because of prodrug metabolism and decrease because of metabolic enzyme reactions. The metabolites are different due to the species of enzymes that metabolize prodrugs. Therefore, the rate at which the metabolic enzyme contributes to the overall metabolism must be considered. The gradient of each metabolite concentration can be expressed by

$$\frac{d[C_{M}]}{dt}=fm\times k_{P}[C_{P}]-k_{M}[C_{M}],$$ (3)

where *km* is the elimination rate constant of the metabolite and *fm* is the fraction metabolized, defined as the metabolized rate by each metabolic enzyme. The change in metabolite concentration then becomes the following:

$$C_{M}[t]=\frac{k_{P}\times fm\times X_{0}}{k_{P}-k_{M}}\times (e^{-k_{M}\times t}-e^{-k_{P}\times t}).$$ (4)

The elimination rate constants can be expressed as

$$k=\frac{CL}{Vd}=\frac{Q\times E}{Vd},$$ (5)

where *CL* is the clearance in the liver part (*μ*l/h), *Vd* is the distribution volume (*μ*l), *Q* is the flow rate in the liver part, and *E* is the extraction ratio in the liver part. The change in metabolite concentration can be expressed as

$$C_{M}[t]=\frac{E_{P}\times fm\times X_{0}}{E_{P}-E_{M}}\times \left(e^{-\frac{Q\times E_{M}}{Vd}\times t}-e^{-\frac{Q\times E_{P}}{Vd}\times t}\right).$$ (6)

A PD model was developed based on the drug efficacy of SN-38, whose concetration was evaluated previously on A549 cells.^17^ SN-38, a metabolite of CPT-11 used in this study, has a strong anticancer effect. The drug efficacy is expressed as

$$\frac{Cell\;density}{Control\;cell\;density}=-0.086\times \ln(AUC)+0.512.$$ (7)

*AUC* (h ng/*μ*l) is an integral part of the concentration change function. Therefore, the drug efficacy after *t* hours from the administration of the drug dose can be expressed as

$$\frac{Cell\;density}{Control\;cell\;density}=-0.086\times \ln\left(\int_{t}^{0}C_{M}[t]\right)+0.512.$$ (8)

### E. Estimating drug efficacy-specific parameters in the PK–PD model

An unknown parameter, the extraction ratio in the liver part *E*, in the PK–PD model can be experimentally estimated using the MOoC. Drug efficacy tests using two different types of the MOoC with different flow rates in the liver part were carried out. CPT-11 (2688, Tocris Bioscience, UK), an anticancer drug widely used in the treatment of alveolus and large intestine cancers, was used as a drug model. The cells were exposed to 15 *μ*m CPT-11 dissolved in the culture medium, which was exchanged every 24 h. After exposure for 72 h, the cells were stained by Hoechst33342 (346-07951, Dojindo Laboratories, Japan) and observed using a CCD camera (DP72, Olympus Corporation, Japan) installed with a fluorescence microscope. The drug effect was evaluated using the density of the A549 cells measured from the fluorescent images. The extraction ratios in the liver part of CPT-11 and SN-38 in the MOoC were estimated from the experimental results using the MOoC with and without bypass channels and our PK–PD model using Eq. (6) with numerical parameters shown in Table I. The numerical parameters, *Vd*, *Q*, and *X_0_*, were determined from the MOoC design and the experimental conditions.

**TABLE I.** Numerical parameters in the PK-PD model to estimate extraction ratios of CPT-11 and SN-38 from the experimental data.

Name	Description	Value	
		w/o bypass channel	w/ bypass channel
*V_d_* (*μ*l)	Distribution volume	70.19	85.01
*Q* (*μ*l/h)	Flow rate in the liver part	89.60	26.45
*X_0_* (ng/*μ*l)	Initial concentration of CPT-11	9.35	9.35
*fm*	Fraction metabolized of CPT-11 by CES2	1	1
$\frac{Cell\;density}{Control\;cell\;density}$	Cell density ratio of A549 cells	0.256	0.332

### F. Evaluation of DDI

A DDI evaluation experiment using the MOoC was carried out using CPT-11 and either simvastatin (S0509, Tokyo Chemical Industry Co., Ltd., Japan), a lipid-lowering drug, or ritonavir (RTV, R0116, Tokyo Chemical Industry Co., Ltd.), an anti-HIV drug, to affect metabolic function changes on the MOoC. The metabolism of CPT-11 to SN-38 would be decreased by the concomitant administration of SV, which is an inhibitor of carboxylesterase 2 (CES2).^18^ We hypothesized that the concomitant administration of CPT-11 and RTV, which is an inhibitor of cytochrome P450 3A4 (CYP3A4), would not affect the amount of SN-38 in the MOoC because CYP3A4 expression in HepG2 cells is quite low.^19^ The CPT-11 concentration was 15 *μ*M, whereas SV concentration was 1 *μ*M, which makes the CES2 expression 50%.^20^ Because the RTV concentration was 10 *μ*M, the CYP3A4 expression of microsomes from the human liver becomes 5%.^21^ The culture medium in the MOoC was exchanged every 24 h. After 72 h, cell densities were observed as previously mentioned. The effects of DDI on pharmacokinetics were evaluated by comparing the predicted cell density of the PK–PD model with that of the experimental results.

### G. Statistical analysis

All values are expressed as mean ± SD of experiments performed in at least triplicates. The unpaired Tukey HSD test was performed for the statistical evaluation, and differences were considered statistically significant when p < 0.05.

## III. RESULTS AND DISCUSSION

### A. Flow characterization of the MOoC

**FIG. 2.**
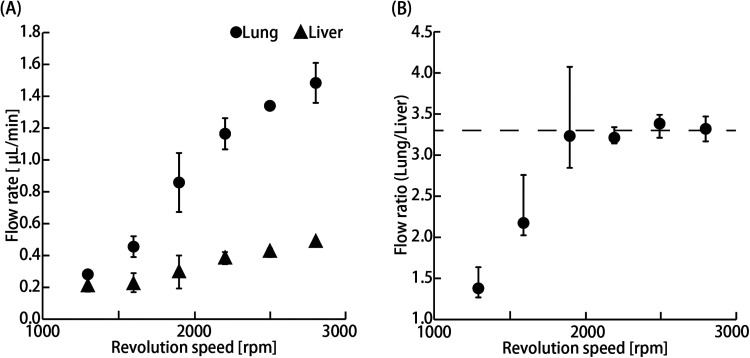
Flow characterization of the MOoC. (a) Flow rate in the liver and lung cancer parts at 1300–2800 rpm (n = 3; mean ± SD) (b) Flow ratio of the lung cancer part to the liver part at 1300–2800 rpm (n = 3; mean ± maximum and minimum). The broken line indicates the physiological flow ratio at approximately 3.3.

The flow rate in the organ parts increased with increasing rotation speed of the stirrer-based micropump [Fig. 2(a)]. The flow ratio was stabilized, and a physiological blood flow ratio of 3.3 was achieved at more than 1900 rpm [Fig. 2(b)].

The increase in flow rate is due to an increase in the amount of liquid pushed out in the same direction as the flow caused by increasing the rotational speed of the stirrer-based micropump. The physiological flow ratio could not be replicated when the stirrer-based micropump rotational speed was less than 1600 rpm, and the dispersion of the flow ratio increased slightly when the speed was less than 1900 rpm. These results suggest that the flow in the MOoC would be stable when the rotational speed of the stirrer-based micropump is above 1900 rpm. A previous study reported that the flow is unsteady when the stirrer-based micropump rotational speed is below 500 rpm, whereas the flow rate increases linearly when the rotation speed is at least 500 rpm.^22^ Our obtained flow rate was lower, whereas the rotational speeds needed to stabilize the flow rate were higher than those of the previous study. The flow from the stirrer-based micropump depends on the flow resistance of a microchannel. The channel's resistance was higher than that of the previous device; therefore, the amount of the liquid pushed out by the stirrer was smaller. The range of the flow rate is limited by the channel resistance, although the stirrer-based micropump is suitable for evaluating organ–organ interactions because the culture medium volume can be reduced, and continuous stable perfusion is possible. Therefore, the rotational speed of the stirrer-based micropump was set at 2800 rpm in consideration of the flow ratio and flow stability in our experiments.

### B. Estimation of the extraction ratio in the liver part by the drug efficacy test using the MOoC

**FIG. 3.**
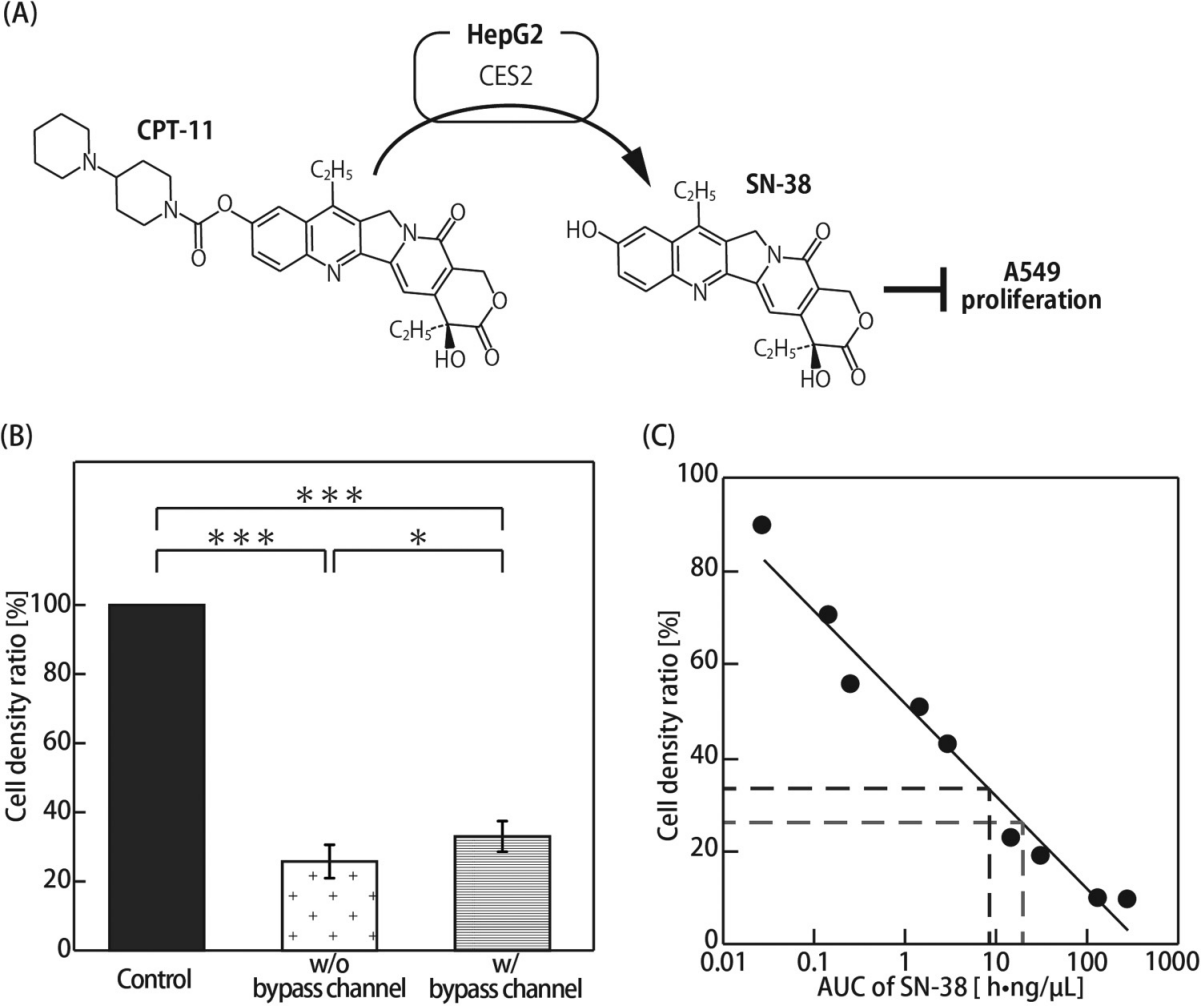
Evaluation of the drug efficacy in the MOoC. (a) Metabolic map of CPT-11 by HepG2. CPT-11 is metabolized to SN-38 by CES2. SN-38 suppresses A549 proliferation. (b) Cell density ratio of A549 in the CPT-11 assay. The cell densities are expressed as the ratio of the cell density to the cell density of the control (n = 3–6; mean ± SD). Asterisks indicate significant differences (* P < 0.05; *** P < 0.005). (c) Cell density ratio as a function of AUC of SN-38. The dark broken line indicates the result of the MOoC with the bypass channel, whereas the light broken line shows the result of the MOoC without the bypass channel.

Two types of the MOoC with and without the bypass channel that have 1.0:3.3 physiological flow ratio (liver:lung cancer part) and 1.0:1.0 non-physiological flow ratio (liver:lung cancer part), respectively, were used to evaluate how the flow ratio influences drug efficacy on the MOoC. A549 cells are widely used as an *in vitro* model of lung cancer to investigate the effects of anticancer drugs. HepG2 cells are not hepatocytes but hepatocellular carcinoma cells; however, HepG2 cells express CES2 at levels comparable to those of human liver microsomes.^23^ Accordingly, we used these cell lines as lung cancer cells and liver model cells to investigate the combination of the PK–PD model and the MOoC. The density ratios of A549 cells exposed to CPT-11 for 72 h on the MOoC with and without bypass channels decreased significantly to 33.2% and 25.6%, respectively, as compared to that of the control, i.e., not exposed to CPT-11, with a significant difference between the MOoC with and without bypass channels [Figs. 3(a) and 3(b)].

Prodrugs are primarily metabolized by metabolic enzymes in the liver, following which, the metabolites distributed by blood flow show the drug effects and side effects on tissues and organs *in vivo*. CPT-11 is metabolized mainly by CES2 and CYP3A4, which are metabolic enzymes in hepatocytes.^24,25^ CPT-11 is metabolized to inactive metabolites (i.e., APC and NPC), which do not have anticancer effects by CYP3A4, whereas it is metabolized to SN-38, which has approximately 1000 times the anticancer effect of CPT-11 by CES2.^26^ SN-38 exhibits a strong anticancer effect by inhibiting the activity of type-1 topoisomerase, which is necessary for cell proliferation.^27^ Moreover, SN-38 is conjugated by UDP-glucuronosyltransferase 1A1 (UGT1A1) to yield SN-38G, which is inert, and thereby has no anticancer activity.^28,29^ The significant decrease in the density ratio of the A549 cells exposed to CPT-11 indicates that CPT-11 was metabolized by the HepG2 cells [Fig. 3(a)], and type-1 topoisomerase was subsequently inhibited by SN-38. The cell density ratio of the MOoC with the bypass channel was significantly higher than that without the bypass channel because the AUC of SN-38 on the MOoC with the bypass channel did not increase due to the decline in clearance, which indicates the ability to remove drugs by metabolism in the liver part with a lower flow rate.

The AUC of SN-38 was obtained from the cell density ratio in the drug efficacy test using the PD model [Eq. (7)]. Then, the extraction ratios of both CPT-11 and SN-38 in the liver part were estimated by the AUC of SN-38 and the PK model [Eq. (6)] (numerical parameters are shown in Table I). The expression level of CYP3A4 in HepG2 is extremely low,^28,29^ and CPT-11 is only metabolized to SN-38 by CES2. Therefore, the *fm_CES2_*, which represents the fraction of metabolism of CPT-11 by CES2, was set to 1. We assumed that the metabolism of SN-38 to SN-38G is irreversible in the PK–PD model because SN-38G does not convert to SN-38 in the MOoC. SN-38G normally changes by conjugation with β-glucuronidase by intestinal bacteria *in vivo*. *Vd* was defined as the volume of the microchannel on the MOoC because the compound does not effuse outside. The AUCs of SN-38 were calculated to be 8.15hng/μl and 19.63hng/μl on the MOoC with and without the bypass channel, respectively [Fig. 3(c)]. The extraction ratios of CPT-11 and SN-38 in the liver part were estimated to be 0.4% and 8.4%, respectively. When human liver microsomes were exposed to CPT-11, SN-38G was generated at approximately 17 times more than the amount of SN-38,^30^ suggesting that the fraction of SN-38 metabolized to SN-38G per unit time is larger than the fraction of CPT-11 metabolized to SN-38 per unit time. The same result was obtained in the HepG2 cells on the MOoC. This result suggested that HepG2 cells still have some potential to assume a part of hepatic functions on the MOoC.

### C. Evaluation of DDI

**FIG. 4.**
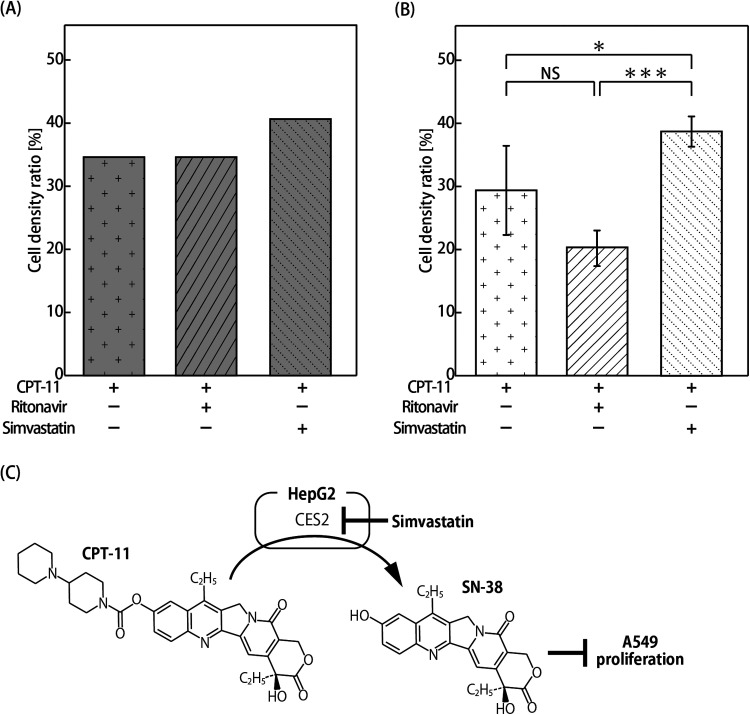
Evaluation of drug efficacy in concomitant administration of metabolic inhibitors. (a) Estimation results using the PK–PD model. The drug efficacies are expressed as ratios of cell density to the cell density of the control condition without CPT-11, TRV, and SV treatment. (b) Cell density ratio of A549 cells in the MOoC expressed as a ratio to the cell density of the control experiment without CPT-11, TRV, and SV treatment (n = 3 or 6; mean ± SD). Asterisks indicate significant differences (* P < 0.05; *** P < 0.005). (c) Metabolic map of CPT-11 by HepG2. CPT-11 is metabolized to SN-38 by CES2. Simvastatin suppresses CES2 activity.

Drug efficacy was predicted using the PK–PD model (the numerical parameters are shown in Table II). The extraction ratio of CPT-11 in the liver part with concomitant administration of SV was set to 0.2% because the CES2 expression would be half.^20^ Meanwhile, the extraction ratio of CPT-11 in the liver part with concomitant administration of RTV was set to 0.4%, which is the same value as that without concomitant administration, because CYP3A4 expression is extremely low in HepG2, and CYP3A4 inhibition might have no effect on pharmacokinetics. The extraction ratio of SN-38 in the liver part was set to 8.4%. A549 cell density ratios after exposure to CPT-11 for 72 h without metabolic inhibitors, with RTV, and with SV were predicted to be 35.5%, 35.5%, and 25.6%, respectively, compared to cells without CPT-11, RTV, and SV treatment (control) [Fig. 4(a)]. Meanwhile, their experimental values using the MOoC were 29.5%, 20.3%, and 38.8%, respectively. The cell density ratio with SV was significantly higher than that without metabolic inhibitors. No statistically significant difference was observed between without metabolic inhibitor and with RTV, although the cell density with RTV was lower than that without the metabolic inhibitor [Fig. 4(b)].

**TABLE II.** Numerical parameters in the PK–PD model to estimate cell density ratios.

Name	Description	Value		
		w/o inhibitors	w/ RTV	w/ SV
*V_d_* (*μ*l)	Distribution volume	85.01	85.01	85.01
*Q* (*μ*l/h)	Flow rate in the liver part	26.45	26.45	26.45
*X_0_* (ng/*μ*l)	Initial concentration of CPT-11	9.35	9.35	9.35
*fm*	Fraction metabolized of CPT-11 by CES2	1	1	1
*E_p_*	Extraction ratio of CPT-11	0.004	0.004	0.002
*E_m_*	Extraction ratio of SN-38	0.084	0.084	0.084

Drug efficacy declined with the concomitant administration of SV because SV suppresses CES2 expression, which metabolizes CPT-11 to SN-38 [Fig. 4(c)]. As expected, drug efficacy did not change with the concomitant administration of RTV, which was used as a CYP3A4 inhibitor, because CYP3A4 expression in HepG2 is quite low. However, drug efficacy with concomitant administration of RTV was markedly increased in the experiment. SN-38 metabolism to SN-38G is decreased by the concomitant administration of anti-HIV drugs, suggesting that anti-HIV drugs impact the metabolism by UGT1A1.^31^ HepG2 cells have low levels of CYP3A4 activity but still express UGT1A1.^32^ The increase in drug efficacy may have been caused by the inhibition of UGT1A1 because the AUC of SN-38 increased by the inhibition of not only CPT-11 metabolism to APC and NPC but also SN-38 metabolism to SN-38G. Our findings involving the DDI of inhibitor chemicals were quite similar to the results of previous studies, indicating that the DDI evaluation using the PK–PD model and OoC is useful.

## IV. CONCLUSION

We reported the DDI estimation method using the PK–PD model based on MOoC and demonstrated its usefulness. We confirmed that the MOoC can be used to evaluate the effect of metabolites on a drug target model from the experimental results using CPT-11. Drug-specific parameters were estimated by a combination of the PK–PD model and the experimental results using the MOoC. Then, DDI estimation methods were evaluated by comparing the calculated drug efficacy of the anticancer drug by the PK–PD model, with experimental results obtained using metabolism inhibitors on the MOoC. The effect of concomitantly administered drugs on the pharmacokinetic changes occurring in the MOoC can be more clearly identified by evaluating DDI by the PK–PD model, using parameters inferred from the experimental results. Our proposed method is useful in evaluating not only liver metabolism but also the DDI effects for different organ functions such as absorption and excretion. In this study, DDI was evaluated only in terms of drug efficacy. For a more in-depth understanding of DDI, evaluating the changes in drug concentration and metabolic capacity through mathematical models and experiments is necessary. The contents of the culture medium and cells in the MOoC should be measured using liquid chromatography–mass spectrometry and PCR for the evaluation. We plan to investigate these aspects in our future studies. Despite this limitation, our method using the MOoC still proved to be valuable for the study and analysis of DDI.

## AUTHORS’ CONTRIBUTIONS

All authors contributed equally to this work.

## Data Availability

The data that support the findings of this study are available within the article.

## References

[1] R. B. Diasio, Br. J. Clin. Pharmacol. 46, 1 (1998). 10.1046/j.1365-2125.1998.00050.x PMC1873978 9690942

[2] J. H. Beijnen and J. H. M. Schellens, Lancet Oncol. 5, 489 (2004). 10.1016/S1470-2045(04)01528-1 15288238

[3] E. M. Dehne, T. Hasenberg, and U. Marx, Future Sci. OA 3, FSO185 (2017). 10.4155/fsoa-2017-0002 28670475 PMC5481853

[4] H. E. Abaci and M. L. Shuler, Integr. Biol. UK 7, 383 (2015). 10.1039/C4IB00292J PMC4407827 25739725

[5] R. Prantil-Baun, R. Novak, D. Das, M. R. Somayaji, A. Przekwas, and D. E. Ingber, Annu. Rev. Pharmacol. Toxicol. 58, 37 (2018). 10.1146/annurev-pharmtox-010716-104748 29309256

[6] S. Ishida, Drug Metab. Pharmacokinet. 33, 49 (2018). 10.1016/j.dmpk.2018.01.003 29398302

[7] E. W. Esch, A. Bahinski, and D. Huh, Nat. Rev. Drug Discovery 14, 248 (2015). 10.1038/nrd4539 25792263 PMC4826389

[8] U. Marx, T. B. Andersson, A. Bahinski, M. Beilmann, S. Beken, F. R. Cassee, M. Cirit, M. Daneshian, S. Fitzpatrick, O. Frey, C. Gaertner, C. Giese, L. Griffith, T. Hartung, M. B. Heringa, J. Hoeng, W. H. De Jong, H. Kojima, J. Kuehnl, A. Luch, D. Sakharov, A. J. A. M. Sips, T. Steger-hartmann, A. Tagle, A. Tonevitsky, T. Tralau, S. Tsyb, A. Van De Stolpe, P. Vulto, J. Wang, J. Wiest, M. Rodenburg, and A. Roth, ALTEX 33 (3), 272–321 (2016). 10.14573/altex.1603161 27180100 PMC5396467

[9] S. N. Bhatia and D. E. Ingber, Nat. Biotechnol. 32, 760 (2014). 10.1038/nbt.2989 25093883

[10] H. Lee, D. S. Kim, S. K. Ha, I. Choi, J. M. Lee, and J. H. Sung, Biotechnol. Bioeng. 114, 432 (2017). 10.1002/bit.26087 27570096

[11] J. H. Sung, C. Kam, and M. L. Shuler, Lab Chip 10, 446 (2010). 10.1039/b917763a 20126684

[12] D. W. Lee, S. H. Lee, N. Choi, and J. H. Sung, Biotechnol. Bioeng. 116, 3433 (2019). 10.1002/bit.27151 31429925

[13] T. Takahashi, *The Atlas of the Human Body* (Kodansha Ltd, Tokyo, 1989).

[14] H. Nakayama, H. Kimura, T. Fujii, and Y. Sakai, J. Biosci. Bioeng. 117, 756 (2014). 10.1016/j.jbiosc.2013.11.019 24374121

[15] J. C. McDonald and G. M. Whitesides, Acc. Chem. Res. 35, 491 (2002). 10.1021/ar010110q 12118988

[16] Y. J. Chuah, S. Kuddannaya, M. H. A. Lee, Y. Zhang, and Y. Kang, Biomater. Sci. 3, 383 (2015). 10.1039/C4BM00268G 26218129

[17] T. Mijatovic, A. O. De Beeck, E. Van Quaquebeke, J. Dewelle, F. Darro, Y. de Launoit, and R. Kiss, Mol. Cancer Ther. 5, 391 (2006). 10.1158/1535-7163.MCT-05-0367 16505114

[18] Y. Shen, Z. Shi, and B. Yan, Nucl. Recept. Res. 6, 101435 (2019). 10.32527/2019/101435

[19] M. Huch, H. Gehart, R. Van Boxtel, K. Hamer, F. Blokzijl, M. M. A. Verstegen, E. Ellis, M. Van Wenum, S. A. Fuchs, J. De Ligt, M. Van De Wetering, N. Sasaki, S. J. Boers, H. Kemperman, J. De Jonge, J. N. M. Ijzermans, E. E. S. Nieuwenhuis, R. Hoekstra, S. Strom, R. R. G. Vries, L. J. W. Van Der Laan, E. Cuppen, and H. Clevers, Cell 160, 299 (2015). 10.1016/j.cell.2014.11.050 25533785 PMC4313365

[20] T. Fukami, S. Takahashi, N. Nakagawa, T. Maruichi, M. Nakajima, and T. Yokoi, Drug Metab. Dispos. 38, 2173 (2010). 10.1124/dmd.110.034454 20810539

[21] V. A. Eagling, D. J. Back, and M. G. Barry, Br. J. Clin. Pharmacol. 44, 190 (1997). 10.1046/j.1365-2125.1997.00644.x 9278209 PMC2042821

[22] H. Kimura, T. Yamamoto, H. Sakai, Y. Sakai, and T. Fujii, Lab Chip 8, 741 (2008). 10.1039/b717091b 18432344

[23] C. C. Wong, K. W. Cheng, G. Xie, D. Zhou, C. H. Zhu, P. P. Constantinides, and B. Rigas, J. Pharmacol. Exp. Ther. 340, 422 (2012). 10.1124/jpet.111.188508 22085648 PMC3263964

[24] R. Mullangi, P. Ahlawat, and N. R. Srinivas, Biomed. Chromatogr. 24, 104 (2010). 10.1002/bmc.1345 19852077

[25] M. K. Ma, W. C. Zamboni, K. M. Radomski, W. L. Furman, V. M. Santana, P. J. Houghton, S. K. Hanna, A. K. Smith, and C. F. Stewart, Clin. Cancer Res. 6, 813 (2000), available at https://clincancerres.aacrjournals.org/content/6/3/813.short. 10741701

[26] K. Uchida, K. Otake, K. Tanaka, K. Hashimoto, S. Saigusa, K. Matsushita, Y. Koike, M. Inoue, M. Ueeda, Y. Okugawa, Y. Inoue, Y. Mohri, and M. Kusunoki, J. Pediatr. Surg. 48, 502 (2013). 10.1016/j.jpedsurg.2012.10.004 23480903

[27] A. Kurita and N. Kaneda, J. Chromatogr. B Biomed. Sci. Appl. 724, 335 (1999). 10.1016/S0378-4347(98)00554-4 10219676

[28] S. Wilkening, F. Stahl, and A. Bader, Drug Metab. Dispos. 31, 1035 (2003). 10.1124/dmd.31.8.1035 12867492

[29] G. Levy, D. Bomze, S. Heinz, S. D. Ramachandran, A. Noerenberg, M. Cohen, O. Shibolet, E. Sklan, J. Braspenning, and Y. Nahmias, Nat. Biotechnol. 33, 1264 (2015). 10.1038/nbt.3377 26501953

[30] J. M. Van Der Bol, W. J. Loos, F. A. De Jong, E. Van Meerten, I. R. H. M. Konings, M. H. Lam, P. De Bruijn, E. A. C. Wiemer, J. Verweij, and R. H. J. Mathijssen, Eur. J. Cancer 47, 831 (2011). 10.1016/j.ejca.2010.11.030 21216137

[31] G. Corona, E. Vaccher, S. Sandron, I. Sartor, U. Tirelli, F. Innocenti, and G. Toffoli, Clin. Pharmacol. Ther. 83, 601 (2008). 10.1038/sj.clpt.6100330 17713471

[32] W. M. A. Westerink and W. G. E. J. Schoonen, Toxicol. In Vitro 21, 1592 (2007). 10.1016/j.tiv.2007.06.017 17716855

